# The relationship between birth intervals and adverse maternal and neonatal outcomes in six low and lower-middle income countries

**DOI:** 10.1186/s12978-020-01008-4

**Published:** 2020-11-30

**Authors:** Melissa Bauserman, Kayla Nowak, Tracy L. Nolen, Jackie Patterson, Adrien Lokangaka, Antoinette Tshefu, Archana B. Patel, Patricia L. Hibberd, Ana L. Garces, Lester Figueroa, Nancy F. Krebs, Fabian Esamai, Edward A. Liechty, Waldemar A. Carlo, Elwyn Chomba, Musaku Mwenechanya, Shivaprasad S. Goudar, Umesh Ramadurg, Richard J. Derman, Sarah Saleem, Saleem Jessani, Marion Koso-Thomas, Elizabeth M. McClure, Robert L. Goldenberg, Carl Bose

**Affiliations:** 1 Department of Pediatrics, University of North Carolina at Chapel Hill, School of Medicine, 101 Manning Drive, Chapel Hill, NC CB 7596 USA; 2 RTI International, Durham, NC USA; 3 Kinshasa School of Public Health, Kinshasa, Democratic Republic of Congo; 4 Lata Medical Research Foundation, Nagpur, India; 5 Boston University School of Public Health, Boston, MA USA; 6 Instituto de Nutrición de Centroamérica y Panamá, Guatemala City, Guatemala; 7 University of Colorado School of Medicine, Denver, CO USA; 8 Moi University School of Medicine, Eldoret, Kenya; 9 Indiana School of Medicine, University of Indiana, Indianapolis, IN USA; 10 University of Alabama at Birmingham, Birmingham, AL USA; 11 University Teaching Hospital, Lusaka, Zambia; 12 KLE Academy Higher Education and Research, J N Medical College Belagavi, Belgaum, Karnataka India; 13 S Nijalingappa Medical College, Bagalkot, Karnataka India; 14 Thomas Jefferson University, Philadelphia, PA USA; 15 Aga Khan University, Karachi, Pakistan; 16 Eunice Kennedy Shriver National Institute of Child Health and Human Development, Bethesda, MD USA; 17 Department of Obstetrics and Gynecology, Columbia University School of Medicine, New York, NY USA

**Keywords:** Birth intervals, Developing countries, Maternal mortality, Neonatal mortality, Low birthweight, Global network

## Abstract

**Background:**

Due to high fertility rates in some low and lower-middle income countries, the interval between pregnancies can be short, which may lead to adverse maternal and neonatal outcomes.

**Methods:**

We analyzed data from women enrolled in the NICHD Global Network Maternal Newborn Health Registry (MNHR) from 2013 through 2018. We report maternal characteristics and outcomes in relationship to the inter-delivery interval (IDI, time from previous delivery [live or stillborn] to the delivery of the index birth), by category of 6–17 months (short), 18–36 months (reference), 37–60 months, and 61–180 months (long). We used non-parametric tests for maternal characteristics, and multivariable logistic regression models for outcomes, controlling for differences in baseline characteristics.

**Results:**

We evaluated 181,782 women from sites in the Democratic Republic of Congo, Zambia, Kenya, Guatemala, India, and Pakistan. Women with short IDI varied by site, from 3% in the Zambia site to 20% in the Pakistan site. Relative to a 18–36 month IDI, women with short IDI had increased risk of neonatal death (RR = 1.89 [1.74, 2.05]), stillbirth (RR = 1.70 [1.56, 1.86]), low birth weight (RR = 1.38 [1.32, 1.44]), and very low birth weight (RR = 2.35 [2.10, 2.62]). Relative to a 18–36 month IDI, women with IDI of 37–60 months had an increased risk of maternal death (RR 1.40 [1.05, 1.88]), stillbirth (RR 1.14 [1.08, 1.22]), and very low birth weight (RR 1.10 [1.01, 1.21]). Relative to a 18–36 month IDI, women with long IDI had increased risk of maternal death (RR 1.54 [1.10, 2.16]), neonatal death (RR = 1.25 [1.14, 1.38]), stillbirth (RR = 1.50 [1.38, 1.62]), low birth weight (RR = 1.22 [1.17, 1.27]), and very low birth weight (RR = 1.47 [1.32,1.64]). Short and long IDIs were also associated with increased risk of obstructed labor, hemorrhage, hypertensive disorders, fetal malposition, infection, hospitalization, preterm delivery, and neonatal hospitalization.

**Conclusions:**

IDI varies by site. When compared to 18–36 month IDI, women with both short IDI and long IDI had increased risk of adverse maternal and neonatal outcomes.

**Trial registration:**

The MNHR is registered at NCT01073475.

## Plain English summary

Due to high fertility rates in some low and lower-middle income countries, the interval between pregnancies can be short, which may lead to poor maternal and neonatal health outcomes. We measured the time between the delivery of one child to the delivery of the next child in six low and lower-middle income countries. We highlight differences, by country, in the number of women who have a short delivery interval from 4% of women in the Zambia site to 20% of women in the Pakistan site. We also highlight differences, by country, in the number of women who have long delivery intervals, from 4% of women in the Democratic Republic of Congo site to 24% of women in the Zambia site. Women with both a short and long delivery interval have higher risk of poor outcomes related to childbirth (obstructed labor, hemorrhage, disorders of high blood pressure, fetal malposition, infection and hospitalization), and poor outcomes for their babies (neonatal death, stillbirth, preterm delivery, low birth weight and hospitalization). Women with long delivery intervals also experience higher risk of maternal death.

## Background

High fertility rates are common in low and lower-middle income countries (LMICs). Among the 6 LMICs included in the *Eunice Kennedy Shriver* National Institute of Child Health and Human Development (NICHD) Global Network for Women’s and Children’s Health Research (GN), fertility rates varied from 2.9 to 6.0 births per woman [1]. High fertility rates lead to shortened time between pregnancies, without allowing the mother to fully recover to baseline health status prior to a subsequent gestation [2, 3]. Short intervals between pregnancies are associated with many adverse health outcomes for the mother, including anemia, placental abruption, placenta previa and uterine rupture [4]. Short birth intervals are also associated with adverse newborn health outcomes such as infant mortality, preterm birth, low birth weight (LBW) and congenital malformations [4–8]. Conversely, long birth intervals can also be associated with adverse maternal and neonatal health outcomes, such as increased risk for induction of labor, chorioamnionitis, Caesarean delivery, preterm birth, LBW, and small for gestational age infants [4, 7, 9]. The ideal timing between pregnancies associated with optimal maternal and neonatal health outcomes has not been definitively established.

The limited existing evidence on the optimal timing between pregnancies is complicated by varying methodologies used to calculate birth spacing. Birth spacing can be defined in several ways, such as the birth-to-pregnancy interval (the period from the prior live birth to the conception of the index pregnancy), the inter-pregnancy interval (the period from the prior birth, regardless of whether the pregnancy resulted in miscarriage/stillbirth/live birth, to the conception of the index pregnancy) or the inter-delivery interval (IDI; the period from the delivery of the prior live birth to the delivery of the index pregnancy) [10]. In the 2005 World Health Organization (WHO) Technical Report, an expert panel preferred birth-to-pregnancy interval to measure birth spacing [10]. Birth-to-pregnancy interval is challenging to measure in low-resource settings where pregnancy dating is inaccurate and therefore length of gestation is difficult to determine [11]. In order to calculate birth-to-pregnancy interval, the expert panel used delivery to delivery interval minus 9 months, thus assuming the index pregnancy resulted in a term gestation. This methodology underestimates the time between births and negates the opportunity to evaluate the effect of birth spacing on the risk of prematurity. The use of IDI might be more appropriate in low-resource settings to investigate associations between birth spacing and neonatal outcomes, without introducing the bias of unknown gestational age.

Based on limited evidence, the WHO recommends a birth-to-pregnancy interval of 24 months, corresponding to an IDI of approximately 33 months, for optimal maternal and neonatal outcomes [10]. After the WHO 2005 Technical Meeting on birth spacing, there was a call for further research to better understand the effect of birth spacing on maternal morbidity and mortality using large datasets. In this paper, we describe IDI in a prospective, multi-country pregnancy registry from 7 research sites in 6 LMICs. We examine maternal characteristics associated with varying lengths of IDI as well as the relationship between adverse delivery and neonatal outcomes and IDI.

## Methods

We analyzed data from women who were enrolled in the NICHD GN’s Maternal Newborn Health Registry (MNHR) from November 2013 through December 2018. The MNHR is a multi-country, population-based, prospectively collected record of pregnancy characteristics as well as maternal and infant outcomes [12]. The MNHR includes research sites in North and South Ubangi, Democratic Republic of Congo (DRC); Kafue and Chongwe (located south and east of the capital city of Lusaka), Zambia; Busia, Bungoma and Kakamega (within the western region), Kenya; Chimaltenango (in the Western Highlands), Guatemala; Belagavi and Bagalkot (within the northern part of the southern state of Karnataka), India; Nagpur (within the state of Maharashtra), India; and Thatta (two of the five sub-districts in the southern Sindh province, near the city of Karachi), Pakistan. The sites represent study clusters from both semi-urban and rural environments.

A detailed description of the MNHR methods are described elsewhere, but briefly, MNHR data were collected from abstraction of medical records as well as a series of interviews conducted by trained study staff [13, 14]. Maternal characteristics, including demographic information, were collected at the time a woman was screened and consented. We also gathered information about the prior pregnancy by maternal report at the time of enrollment. Antenatal and delivery characteristics were recorded within 3–7 days after delivery. Postpartum characteristics were collected at a clinic or home visit 6 weeks after delivery. Maternal anthropometry was not routinely collected at all sites throughout the study period. Maternal weight was collected at the time of enrollment, however enrollment could occur at any time during pregnancy, so these measurements do not provide a consistent reflection of nutritional status. Maternal height was collected at most sites, but this measurement was not collected until 2017 in Kenya. Body mass index (BMI) was calculated from maternal height and weight, when available. Due to these methodological limitations, maternal anthropometry is presented as descriptive data only.

In this analysis, we included all women in the MNHR with an index pregnancy during the specified time period with the following exclusions: women who were lost to follow up prior to delivery, primiparous women, women without a previous pregnancy lasting greater than or equal to 20 weeks, women with unknown parity, multiparous women who had a missing or unknown delivery date for the previous pregnancy, women whose index pregnancy resulted in a miscarriage or medically terminated pregnancy (MTP), and women who had an extreme IDI (< 6 months or > 180 months (15 years)). We defined miscarriage or MTP as a pregnancy that ended prior to 20 weeks gestation. As we could not reliably collect data on pregnancies resulting in a miscarriage or MTP across all sites, these pregnancies were also excluded.

We evaluated IDI in 4 categories based on distinctions in the medical literature: 6–17 months, 18–36 months, 37–60 months, > 60 months [9]. We defined short IDI as the interval from 6 to 17 months. We chose 6 months as the lower limit for analysis to account for at least a 1-month period for return to fecundability and an additional 5 months gestation, since we excluded index pregnancies that resulted in miscarriage or MTP before 20 weeks / 5 months gestation. The category 18–36 months includes the WHO recommended 33 months for optimal birth spacing and therefore was used as the referent category. We defined long IDI as > 60 months. We calculated IDI as the number of months from the date of delivery of the previous pregnancy (resulting in a liveborn or stillborn infant), as reported by the mother, to the date of delivery of the index pregnancy.

To determine if the distribution of IDI differed across sites, we performed a non-parametric Kruskal Wallis test for overall site difference and non-parametric Wilcoxon rank sum tests for all pairwise site comparisons. To determine the relationship between IDI and maternal characteristics, we performed Cochran-Mantel-Haenszel tests of each maternal factor and IDI category stratified by cluster. Risk of maternal outcomes and fetal/neonatal outcomes associated with IDI categories were determined from multivariable generalized linear models with general estimating equations to control for cluster level effects. Models were adjusted for maternal age, education, parity, antenatal care (ANC) visits and iron supplementation. In the maternal risk factors model, the mode of delivery was not included because it is not solely a maternal risk factor and is often influenced by the occurrence of several of the other outcomes. Maternal height, weight, and BMI were not included due to poor data consistency. We used a Poisson distribution for the low-prevalence outcomes of stillbirth and very low birth weight (VLBW), all other outcomes were modeled with a binomial distribution. We report the relative risks (RR) and 95% confidence intervals (CIs) for each outcome by IDI categories with the referent category of 18–36 months. Fetal/neonatal outcomes are reported at the maternal level if at least one fetus/neonate from a multiple birth pregnancy had that outcome.

At each site, institutional review boards or research ethics committees and Ministries of Health approved the collection of data included in the MNHR. We used sensitization meetings to achieve approval within local communities prior to the initiation of the study. All study participants were enrolled with informed consent. A data monitoring committee appointed by the NICHD oversaw and reviewed the MNHR annually.

## Results

We screened 314,313 pregnant women in 7 research sites for inclusion in the MNHR from November 2013 through 2018 (Table 1). We included 312,885 (99.5%) who were eligible and consented. After exclusion of women with unknown parity and nulliparous women, we retained 213,198 (68.1%) women. After exclusions for loss to follow-up prior to delivery, MTP, unreliable prior delivery date and IDI outlying the desired range, we included 181,782 (58.1%) women for analysis. Sites differed in the number of nulliparous mothers (17.1% in the Pakistan site to 49.2% in the Nagpur site). Of the 181,782 subjects, each site contributed between 20,148 and 34,342 women for analysis (Table 2). The distribution of IDI differed across sites overall as well as for each pairwise site comparison (*p* < 0.0001 for all comparisons). The percentage of women with short IDI varied from 3.4% of women in the Zambia site to 19.8% of women in the Pakistan site. The percentage of women with long IDI varied from 4.1% of women in the DRC site to 23.9% of women in the Zambia site. The overall median IDI was 32 (24, 45) months, ranging from 27 months in the Belagavi and Pakistan sites to 43 months in the Zambia site.

**Table 1:** Derivation of Study Population^a^

	Total	DRC	Zambia	Kenya	Guatemala	Belagavi	Nagpur	Pakistan
Screened	314,313	32,449	36,276	40,545	57,247	51,226	47,857	48,713
Ineligible	655	0	0	0	11	0	0	644
Did not consent	773	0	0	3	757	12	0	1
Parity unknown	2686	2	8	111	2	3	30	2530
Parity = 0	97,001	6118	11,127	13,165	16,884	18,390	23,513	7804
Lost to follow-up prior to delivery	1979	415	109	398	292	3	60	702
Last delivery date missing or unknown	18,146	606	2061	1592	4370	2774	2533	4210
Miscarriage/MTP on index pregnancy	10,558	215	101	104	365	5084	1514	3175
Extreme Outlier IDI^b^	733	36	95	54	134	165	59	190
Deliveries Included	181,782	25,057	22,775	25,118	34,432	24,795	20,148	29,457

^a^Includes deliveries and expected deliveries from November 2013 to December 2018

^b^IDI’s < 6 months or > 180 months (15 years) were excluded from this analysis due to validity concerns

**Table 2:** Description of IDI by Site

	Total	DRC	Zambia	Kenya	Guatemala	Belagavi	Nagpur	Pakistan
Deliveries, N	181,782	25,057	22,775	25,118	34,432	24,795	20,148	29,457
IDI Categories, N (%)								
6–17 months	17,392 (9.6)	1712 (6.8)	774 (3.4)	1704 (6.8)	3233 (9.4)	2589 (10.4)	1546 (7.7)	5834 (19.8)
18–36 months	91,670 (50.4)	15,937 (63.6)	7401 (32.5)	10,197 (40.6)	16,046 (46.6)	15,524 (62.6)	10,786 (53.5)	15,779 (53.6)
37–60 months	50,447 (27.8)	6389 (25.5)	9150 (40.2)	8669 (34.5)	8749 (25.4)	5044 (20.3)	5896 (29.3)	6550 (22.2)
61–180 months	22,273 (12.3)	1019 (4.1)	5450 (23.9)	4548 (18.1)	6404 (18.6)	1638 (6.6)	1920 (9.5)	1294 (4.4)
IDI Summary Statistics^a^								
Min-Max	6–180	6–175	6–180	6–179	6–180	6–176	6–180	6–176
Median (P25-P75)	32 (24, 45)	31 (25, 38)	43 (32, 59)	38 (27, 53)	33 (23, 52)	27 (22, 38)	32 (24, 43)	27 (19, 37)
Mean (std)	37.9 (21.7)	33.3 (14.1)	48.7 (24.5)	43.4 (23.6)	41.7 (26.8)	32.4 (17.6)	36.5 (18.8)	29.7 (14.9)

^a^The Kruskal Wallis test for overall location difference across sites has a *p* < 0.0001. All pairwise site differences have Wilcoxon rank sum test *p*-values < 0.0001

**Fig. 1:**
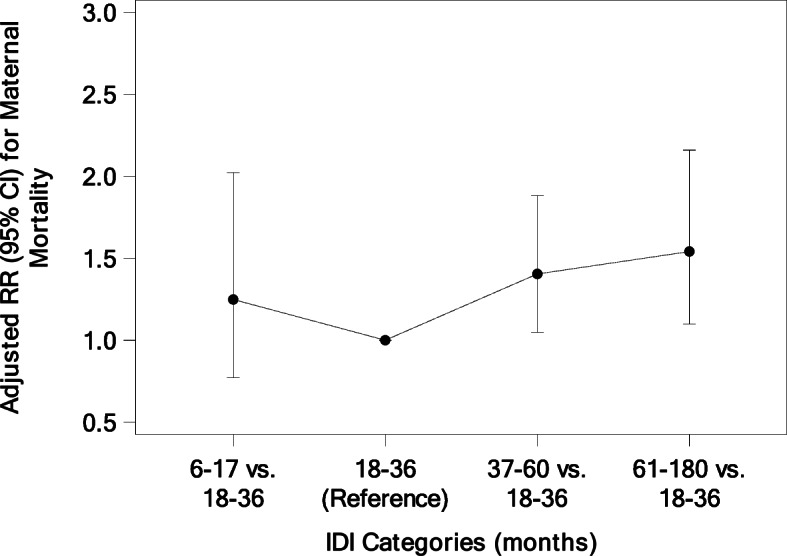
Adjusted Relative Risk for Maternal Mortality < 42 Days by IDI Categories (months)

All maternal characteristics showed a statistically significant difference when evaluated by IDI (Table 3, *p* < 0.001 for all comparisons). Generally, as IDI lengthened, women were more likely to be older, more educated, receive more ANC and receive more iron supplements. Descriptive data for maternal anthropometry is included in the supplemental material. In the multivariable models, short and long IDI had significantly greater risk for nearly all adverse maternal outcomes when compared to an 18–36-month IDI (Table 4). Women with a short IDI had an increased risk of obstructed labor (RR = 1.17 [1.07, 1.28]), maternal hemorrhage (RR = 1.17 [1.04, 1.33]), hypertensive disorders (RR = 1.38 [1.19,1.61]), fetal malposition (RR = 1.27 [1.11, 1.46]), maternal infection (RR = 1.35 [1.17, 1.56]) and maternal hospitalization (RR = 1.31 [1.22, 1.41]). Women with a long IDI also had increased risk of the same adverse delivery outcomes: obstructed labor (RR = 1.54 [1.43, 1.65]), maternal hemorrhage (RR = 1.19 [1.06, 1.32]), hypertensive disorders (RR = 2.10 [1.87, 2.36]), fetal malposition (RR = 1.34 [1.22, 1.48]), maternal infection (RR = 1.33 [1.21, 1.46]), and maternal hospitalization (RR = 1.55 [1.43, 1.67]). Additionally, women with a long IDI had an increased risk of maternal mortality (RR = 1.54 [1.10, 2.16]). Overall, the adjusted RR for maternal mortality was increased for all IDI categories relative to the 18–36-month referent group, although only statistically significant for 37–60 months and long IDI (Fig. 1).

**Table 3:** Maternal Factors and Delivery Mode Associated with IDI^a^

	IDI (months)			
	6–17 months	18–36 months	37–60 months	61–180 months
Deliveries, N	17,392	91,670	50,447	22,273
Maternal age, N (%)	17,388	91,644	50,439	22,270
< 20	1324 (7.6)	4493 (4.9)	1200 (2.4)	81 (0.4)
20–35	15,215 (87.5)	81,857 (89.3)	45,070 (89.4)	18,452 (82.9)
> 35	849 (4.9)	5294 (5.8)	4169 (8.3)	3737 (16.8)
Education, N (%)	17,388	91,649	50,444	22,268
No formal education	6607 (38.0)	26,757 (29.2)	11,530 (22.9)	3151 (14.2)
Primary	3823 (22.0)	23,147 (25.3)	11,796 (23.4)	6162 (27.7)
Secondary	6330 (36.4)	38,671 (42.2)	24,628 (48.8)	11,439 (51.4)
University+	628 (3.6)	3074 (3.4)	2490 (4.9)	1516 (6.8)
Parity, N (%)	17,392	91,670	50,447	22,273
1	7520 (43.2)	36,351 (39.7)	19,353 (38.4)	7441 (33.4)
2	3437 (19.8)	20,123 (22.0)	11,350 (22.5)	5321 (23.9)
≥ 3	6435 (37.0)	35,196 (38.4)	19,744 (39.1)	9511 (42.7)
Number of ANC visits, N (%)	17,376	91,541	50,396	22,243
0	708 (4.1)	2821 (3.1)	1057 (2.1)	351 (1.6)
1–3	8167 (47.0)	39,699 (43.4)	20,258 (40.2)	8300 (37.3)
≥ 4	8501 (48.9)	49,021 (53.6)	29,081 (57.7)	13,592 (61.1)
Iron supplements, N (%)	17,390	91,644	50,436	22,267
Yes	15,010 (86.3)	83,671 (91.3)	47,320 (93.8)	21,423 (96.2)
Mode of delivery, N (%)	17,381	91,619	50,416	22,266
Vaginal/Vaginal assisted	15,064 (86.7)	81,273 (88.7)	44,127 (87.5)	18,580 (83.4)
C-section	2317 (13.3)	10,346 (11.3)	6289 (12.5)	3686 (16.6)

^a^All maternal factors have *p*-values < 0.001 for Cochran–Mantel–Haenszel tests of each maternal factor and IDI stratified by cluster

**Table 4:** Risk of Adverse Delivery Outcomes Associated with IDI

	IDI (months)^a^				Adjusted RR (95% CI)^b^		
	6–17 N (%)	18–36 N (%)	37–60 N (%)	61–180 N (%)	6–17 vs. 18–36	37–60 vs. 18–36	61–180 vs. 18–36
Deliveries	17,392	91,670	50,447	22,273			
Obstructed labor	711 (4.1)	2858 (3.1)	1765 (3.5)	1007 (4.5)	1.17 (1.07, 1.28)	1.18 (1.11, 1.24)	1.54 (1.43, 1.65)
Maternal hemorrhage	525 (3.0)	1891 (2.1)	955 (1.9)	458 (2.1)	1.17 (1.04, 1.33)	1.01 (0.93, 1.10)	1.19 (1.06, 1.32)
Hypertension/pre-eclampsia/eclampsia	359 (2.1)	1242 (1.4)	916 (1.8)	731 (3.3)	1.38 (1.19, 1.61)	1.39 (1.26, 1.54)	2.10 (1.87, 2.36)
Fetal malposition	396 (2.3)	1419 (1.5)	773 (1.5)	491 (2.2)	1.27 (1.11, 1.46)	1.03 (0.95, 1.13)	1.34 (1.22, 1.48)
Maternal infection	375 (2.3)	1217 (1.4)	625 (1.4)	323 (1.6)	1.35 (1.17, 1.56)	1.05 (0.96, 1.16)	1.33 (1.21, 1.46)
Maternal hospitalization	1109 (7.1)	4724 (5.7)	3006 (6.6)	2080 (10.1)	1.31 (1.22, 1.41)	1.17 (1.11, 1.23)	1.55 (1.43, 1.67)
Maternal death < 42 days, N (Rate/100,000 deliveries)	35 (202)	139 (152)	94 (187)	40 (180)	1.25 (0.77, 2.02)	1.40 (1.05, 1.88)	1.54 (1.10, 2.16)

^a^Columns present N (%) for each adverse delivery outcome within each IDI category with the exception of maternal death < 42 days which is presented as rate/100,000 deliveries

^b^Relative risks and 95% confidence intervals from a logistic model with generalized estimating equations to account for the correlation of outcomes within cluster adjusting for maternal age, education, parity, ANC visits, and iron supplements. All Maternal outcomes used a Binomial distribution

**Fig. 2:**
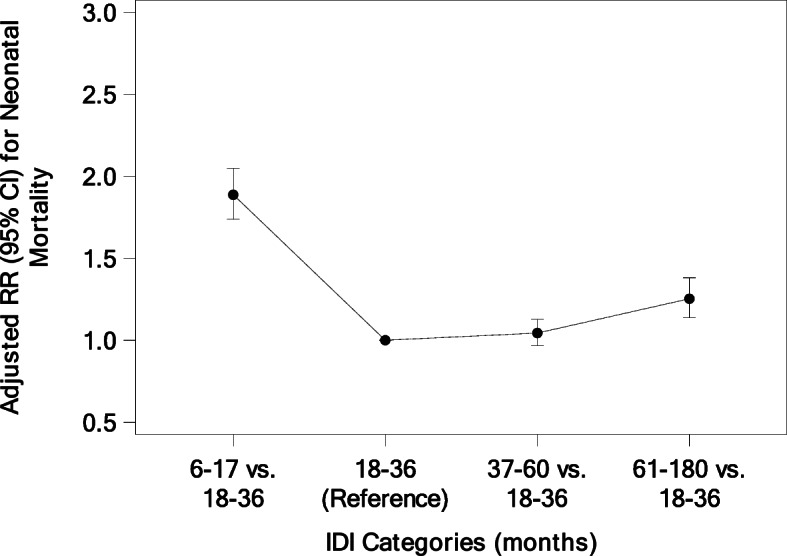
Adjusted Relative Risk for Neonatal Mortality < 28 Days by IDI Categories (months)

**Fig. 3:**
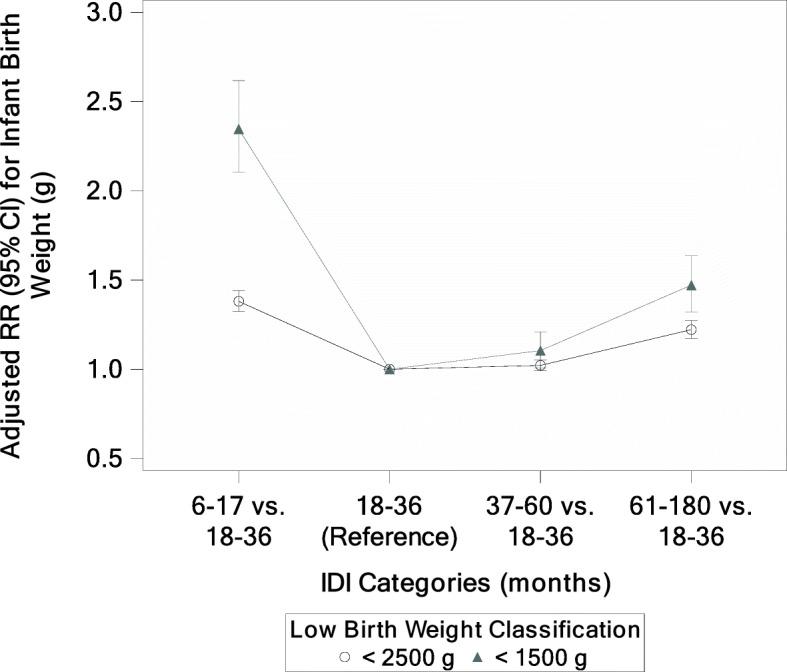
Adjusted Relative Risk for Stillbirth by IDI Categories (months)

**Fig. 4:**
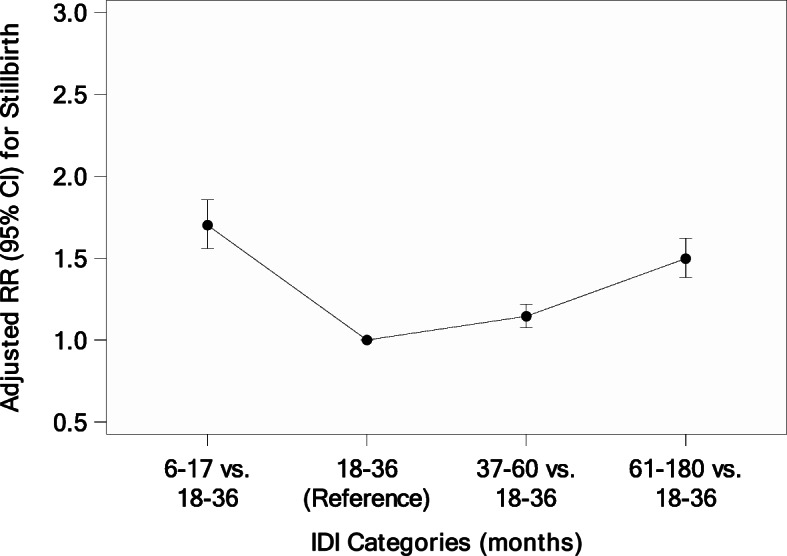
Adjusted Relative Risk for Low Birth Weight by IDI Categories (months)

Similarly, short and long IDI had significantly greater risk for nearly all adverse fetal/neonatal outcomes when compared to an 18–36-month IDI (Table 5). Women with a short IDI had an increased risk of LBW (RR = 1.38 [1.32, 1.44]) and VLBW (RR = 2.35 [2.10, 2.62]), stillbirth (RR = 1.70 [1.56, 1.86]), neonatal mortality (RR = 1.89 [1.74, 2.05]), preterm delivery (RR = 1.44 [1.39, 1.50]), and neonatal hospitalization (RR = 1.24 [1.11, 1.38]). Women with a long IDI also had increased risk of the same adverse neonatal outcomes: LBW (RR = 1.22 [1.17, 1.27]) and VLBW (RR = 1.47 [1.32,1.64]), stillbirth (RR = 1.50 [1.38, 1.62]), neonatal mortality (RR = 1.25 [1.14, 1.38]), preterm delivery (RR = 1.06 [1.02, 1.10]), and neonatal hospitalization (RR = 1.28 [1.15, 1.43]). Overall, the adjusted RR for neonatal mortality was increased for all IDI categories relative to the 18–36-month referent group, although the increase at 37–60 months was very small and not statistically significant (Fig. 2). In contrast, the adjusted RR for stillbirth and very low birth weight was statistically significantly increased for all IDI categories relative to the 18–36-month referent group (Figs. 3 and 4).

**Table 5:** Risk of Adverse Neonatal Outcomes Associated with IDI^a^

	IDI (months)^b^				Adjusted RR (95% CI)^c^		
	6–17 N (%)	18–36 N (%)	37–60 N (%)	61–180 N (%)	6–17 vs. 18–36	37–60 vs. 18–36	61–180 vs. 18–36
Deliveries, N	17,392	91,670	50,447	22,273			
LBW (< 2500 g)	3705 (21.4)	12,643 (13.8)	5941 (11.8)	2864 (12.9)	1.38 (1.32, 1.44)	1.02 (0.99, 1.05)	1.22 (1.17, 1.27)
VLBW (< 1500 g)	572 (3.3)	1128 (1.2)	567 (1.1)	296 (1.3)	2.35 (2.10, 2.62)	1.10 (1.01, 1.21)	1.47 (1.32, 1.64)
Preterm	3934 (22.7)	13,067 (14.3)	6403 (12.8)	2789 (12.6)	1.44 (1.39, 1.50)	0.97 (0.94, 1.00)	1.06 (1.02, 1.10)
Congenital anomalies	36 (0.2)	166 (0.2)	94 (0.2)	42 (0.2)	1.08 (0.78, 1.50)	1.13 (0.88, 1.45)	1.11 (0.78, 1.59)
Neonatal hospitalization	378 (2.5)	1518 (1.9)	742 (1.7)	470 (2.4)	1.24 (1.11, 1.38)	1.02 (0.94, 1.12)	1.28 (1.15, 1.43)
Stillbirth, N (Rate/1000)	792 (45.6)	2219 (24.2)	1246 (24.7)	644 (28.9)	1.70 (1.56, 1.86)	1.14 (1.08, 1.22)	1.50 (1.38, 1.62)
Neonatal death < 28 days, N (Rate/1000)	731 (44.1)	1848 (20.7)	929 (18.9)	456 (21.1)	1.89 (1.74, 2.05)	1.04 (0.97, 1.13)	1.25 (1.14, 1.38)

^a^Fetal/Neonatal outcomes are calculated at the maternal level if at least one fetus/neonate has the outcome

^b^Columns present N (%) for each adverse delivery outcome within each IDI category, with the exception of stillbirth and neonatal death < 28 days which are presented as rate/1000 deliveries

^c^Relative risks and 95% confidence intervals from a logistic model with generalized estimating equations to account for the correlation of outcomes within cluster adjusting for maternal age, maternal education, parity, ANC visits, and iron supplements. Very low birthweight (< 1500) and Stillbirth outcomes used a Poisson distribution, all other Fetal/Neonatal outcomes used a Binomial distribution

## Discussion

Our results indicate that IDI is associated with a number of adverse maternal and neonatal health consequences. We highlight differences by country in the number of women who have a short IDI, from 3% of women in the Zambia site to 20% of women in the Pakistan site. We also highlight differences by country in the number of women who have a long IDI, from 4% of women in the Pakistan and DRC sites to 24% of women in the Zambia site. Women with both a short and long IDI have higher risk of adverse delivery outcomes (obstructed labor, hemorrhage, hypertensive disorders, fetal malposition, infection and hospitalization) and adverse neonatal outcomes (neonatal death, stillbirth, preterm delivery, LBW, VLBW and hospitalization). Women with a long IDI also experience higher risk of maternal death.

Similar to other studies, we demonstrated a bimodal distribution of adverse birth outcomes, with increased risk among women with short and long IDI [15–17]. Of particular note, we demonstrated an increased risk of LBW and VLBW infants at the extremes of IDI. For short birth intervals, this might be explained by a nutritional depletion hypothesis in which the short birth interval results from maternal nutrient deficiency after depletion from the previous gestational period, particularly folate deficiency, which results in impaired fetal growth [2, 18]. However, long IDIs were also associated with LBW infants. This finding indicates that the nutritional depletion hypothesis is insufficient to explain LBW among this group. Among women with a long IDI, we observed a higher RR of hypertensive disorders. The higher prevalence of hypertensive disorders, such as pre-eclampsia, might be a potential mechanism leading to LBW infants, given the established causal relationship in which pre-eclampsia leads to prematurity and growth restriction [19].

The findings of our study support the WHO recommendations for an optimal IDI of 33 months. When compared to short and long intervals, the interval around 33 months was associated with the best maternal and neonatal outcomes. We evaluated IDI categorically in order to compare with previous studies and evaluate current recommendations. Analysis by categories is advantageous given the nonlinear relationship with delivery intervals and birth outcomes [20]. While this approach did not allow for prediction of the optimal number of months for IDI, our robust data support the WHO recommendations of IDI of approximately 33 months.

We noted important differences in maternal characteristics by IDI. Women who had longer IDIs also had characteristics that are usually associated with better delivery outcomes, for example older age, more education, the receipt of more ANC, and receipt of iron supplements. Before we adjusted for these associations, it appeared that neonatal outcomes improved with increasing IDI. However, when we adjusted for maternal characteristics in our models, the models showed an increased risk of adverse neonatal outcomes at both lower and higher IDI categories. This indicates that women with long IDIs do not return to baseline risk for adverse perinatal outcomes even with improvements in health seeking behaviors.

Our study had a number of strengths. The MNHR pregnancy cohort includes a large and multi-national cohort of women from Africa, Asia and Central America. Our data were collected prospectively and included all pregnant women within a study community, allowing population level conclusions within those communities. Our dataset allowed us to describe associated maternal risk factors in addition to both maternal and neonatal outcomes within this population. However, we were also limited by some of the characteristics of the MNHR. We were limited in our ability to assess maternal nutritional status, therefore we are limited in our ability to address the nutrition hypotheses that might contribute to LBW associated with IDI. Because our studies occurred in low-resource settings, there might have been some variability in the reliable assignment of gestational age that could have introduced bias in our results if small for gestational age infants born at term were assigned to the premature category. To improve interpretation of birth weight, we chose to also evaluate LBW and VLBW separately. We chose to evaluate IDI rather than inter-pregnancy interval, so pregnancies that did not last more than 20 weeks are not included. Therefore, our analyses are limited since these pregnancies contribute to adverse maternal and neonatal outcomes.

While the social factors that determine birth spacing are complex and include familial influences and community level influences, there have been some intervention strategies that have been successful in lengthening birth intervals and mitigating some of the risk of adverse maternal and neonatal health outcomes [21, 22]. For example, in Bangladesh, a package of family planning interventions integrated into maternal and newborn health visits decreased the number of women who had a subsequent short birth interval and lowered the risk of preterm birth [23].

## Conclusions

Our data increase the body of literature describing optimal birth intervals in relationship to maternal and newborn health outcomes in LMICs. We describe increased health risk at extremes of birth intervals and support the WHO recommendations for optimal birth spacing. Our data illustrate geographical differences in IDI which underpin the need for programmatic public health efforts to improve birth spacing in certain areas to achieve optimal maternal and neonatal outcomes. These data can inform communities with high rates of sub-optimal birth spacing to direct public health strategies to the regions in most need.

## Supplementary information

**Additional file 1.**

## Data Availability

The datasets generated and analysed during the current study are not yet publicly available due to ongoing data analyses, but they will be available in the NICHD Data and Specimen Hub. Requests for data prior to the public release will be handled by the authors.

## Abbreviations

IDI: Interval delivery interval
LMIC: Low and lower-middle income countries
NICHD: National Institute of Health *Eunice Kennedy Shriver National* Institute of Child Health and Human Development
GN: NICHD Global Network for Women’s and Children’s Health Research
MNHR: Maternal Newborn Health Registry
LBW: Low birth weight
VLBW: Very low birth weight
WHO: World Health Organization
DRC: Democratic Republic of Congo
BMI: Body Mass Index
MTP: Medical termination of pregnancy
RR: Relative Risks
CI: Confidence Intervals
ANC: Antenatal care
