# Supplementary material for: The relationship between birth intervals and adverse maternal and neonatal outcomes in six low and lower-middle income countries
Source: Reprod Health. 2020 Nov 30;17(Suppl 2):157. doi: 10.1186/s12978-020-01008-4 (PMC7708104; doi:10.1186/s12978-020-01008-4)
Supplement: Supplementary file 1 — Additional file 1. [file 12978_2020_1008_MOESM1_ESM.docx]

**Supplementary Table**^1^

|  | Total | DRC | Zambia | Kenya | Guatemala | Belagavi | Nagpur | Pakistan |
| --- | --- | --- | --- | --- | --- | --- | --- | --- |
| Deliveries, N | 181,782 | 25,057 | 22,775 | 25,118 | 34,432 | 24,795 | 20,148 | 29,457 |
| Maternal Height (cm)^2^ | 165,924 | 25,056 | 22,774 | 9,570 | 34,189 | 24,778 | 20,147 | 29,410 |
| Mean (std) | 153.7 (7.3) | 157.1 (6.8) | 158.5 (6.6) | 160.7 (6.5) | 146.7 (5.4) | 152.3 (5.5) | 152.2 (5.4) | 154.9 (5.6) |
| Maternal Weight (kg)^3^ | 177,492 | 25,055 | 22,774 | 21,013 | 34,329 | 24,772 | 20,141 | 29,408 |
| Mean (std) | 53.81 (10.66) | 53.14 (7.21) | 60.86 (10.63) | 61.97 (9.34) | 57.16 (9.81) | 47.40 (7.87) | 45.88 (7.63) | 50.00 (9.79) |
| BMI (kg/m^2^)^4^ | 165,850 | 25,054 | 22,773 | 9,560 | 34,159 | 24,768 | 20,139 | 29,397 |
| Mean (std) | 22.570 (4.299) | 21.513 (2.396) | 24.230 (4.017) | 24.048 (3.315) | 26.510 (4.092) | 20.432 (3.200) | 19.810 (3.142) | 20.818 (3.808) |

^1^ All pairwise IDI category differences for height, weight, and BMI are statistically different (Wilcoxon rank sum test p-values <0.0001). All pairwise site differences for height, weight, and BMI are statistically different (Wilcoxon rank sum test p-values <0.05).

^2^ Kenya did not regularly collect maternal height until 2017.

^3^ Maternal weight is collected at enrollment, which can be any time during pregnancy, including after delivery.

^4^ BMI (body mass index) is derived from maternal height and maternal weight, as such, BMI is also subject to the limitations noted in footnotes 2 and 3 above.
